# Health Effects Associated With Pre- and Perinatal Exposure to Arsenic

**DOI:** 10.3389/fgene.2021.664717

**Published:** 2021-09-29

**Authors:** Victor D. Martinez, Wan L. Lam

**Affiliations:** ^1^ Department of Pathology, Dalhousie University, Halifax, NS, Canada; ^2^ Department of Pathology and Laboratory Medicine, IWK Health Centre, Halifax, NS, Canada; ^3^ Beatrice Hunter Cancer Research Institute, Halifax, NS, Canada; ^4^ The Canadian Environmental Exposures in Cancer (CE2C) Network, Halifax, NS, Canada; ^5^ Department of Integrative Oncology, BC Cancer Research Institute, Vancouver, BC, Canada

**Keywords:** arsenic, DNA methylation, *in utero* exposure, fetal development, genetics, epigenetics

## Abstract

Inorganic arsenic is a well-established human carcinogen, able to induce genetic and epigenetic alterations. More than 200 million people worldwide are exposed to arsenic concentrations in drinking water exceeding the recommended WHO threshold (10μg/l). Additionally, chronic exposure to levels below this threshold is known to result in long-term health effects in humans. The arsenic-related health effects in humans are associated with its biotransformation process, whereby the resulting metabolites can induce molecular damage that accumulates over time. The effects derived from these alterations include genomic instability associated with oxidative damage, alteration of gene expression (including coding and non-coding RNAs), global and localized epigenetic reprogramming, and histone posttranslational modifications. These alterations directly affect molecular pathways involved in the onset and progression of many conditions that can arise even decades after the exposure occurs. Importantly, arsenic metabolites generated during its biotransformation can also pass through the placental barrier, resulting in fetal exposure to this carcinogen at similar levels to those of the mother. As such, more immediate effects of the arsenic-induced molecular damage can be observed as detrimental effects on fetal development, pregnancy, and birth outcomes. In this review, we focus on the genetic and epigenetic damage associated with exposure to low levels of arsenic, particularly those affecting early developmental stages. We also present how these alterations occurring during early life can impact the development of certain diseases in adult life.

## Introduction

Exposure to arsenic in drinking water has health effects in humans, including disorders of the skin, alterations in the vascular/respiratory systems, and cancer (World Health Organization, 2004; Podgorski and Berg, 2020). The WHO has recommended that the levels of arsenic in drinking water should not exceed 10μg/l (Gorchev and Ozolins, 1984). Worldwide, ~220 million people are potentially exposed to high arsenic concentrations (Podgorski and Berg, 2020).

**Figure 1:**
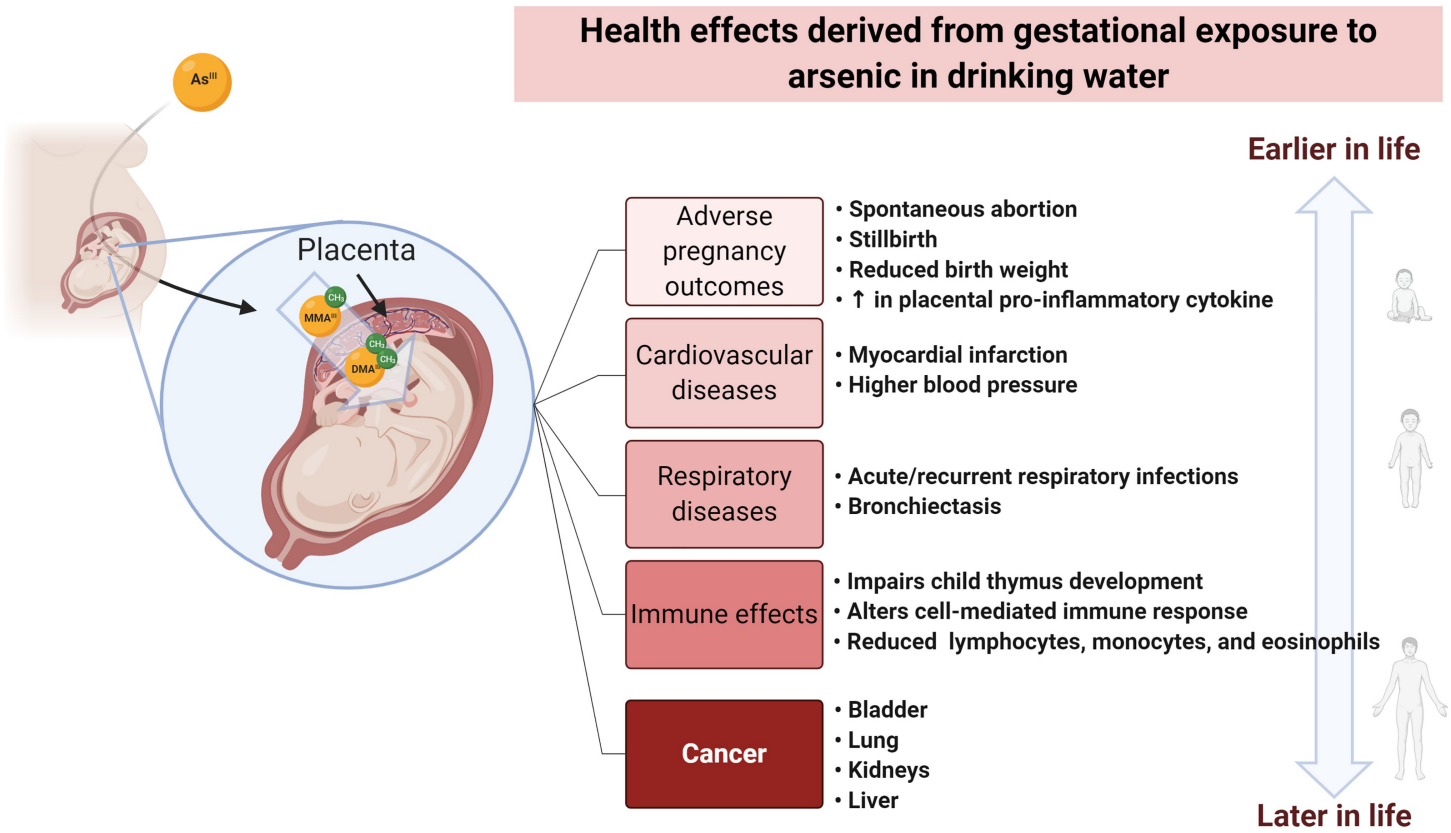
Health effects derived from gestational exposure to arsenic in drinking water. Arsenic ingested by the mother can cross the placental barrier. Gestational exposure to arsenic has been associated with effects at pre-and perinatal stages, as well as during childhood. These effects include adverse pregnancy outcomes and alterations to the cardiovascular, respiratory, and immune system. Moreover, *in utero* exposure has been correlated with an increased risk of developing cancer and other types of diseases during the adult life. Created with BioRender.com.

Genetic and epigenetic alterations have been associated with exposure to arsenic (Figure 1). Damage at the genetic level is associated with the generation of reactive oxygen species (ROS), while epigenetic effects are linked with arsenic metabolism. Gestational (*in utero*) exposure to arsenic has been also associated with health effects arising in pre- and perinatal stages as well as during childhood, as well as an increased risk of developing diseases during adulthood (Naujokas et al., 2013; Quansah et al., 2015; Howard, 2018; Navas-Acien et al., 2019; Salmeri et al., 2020). An increasing number of publications illustrates the recent interest in the association between prenatal exposure and health effects (Supplementary Figure 1).

## Arsenic Metabolism is Associated with an Increased Damaging Potential

**Figure 2:**
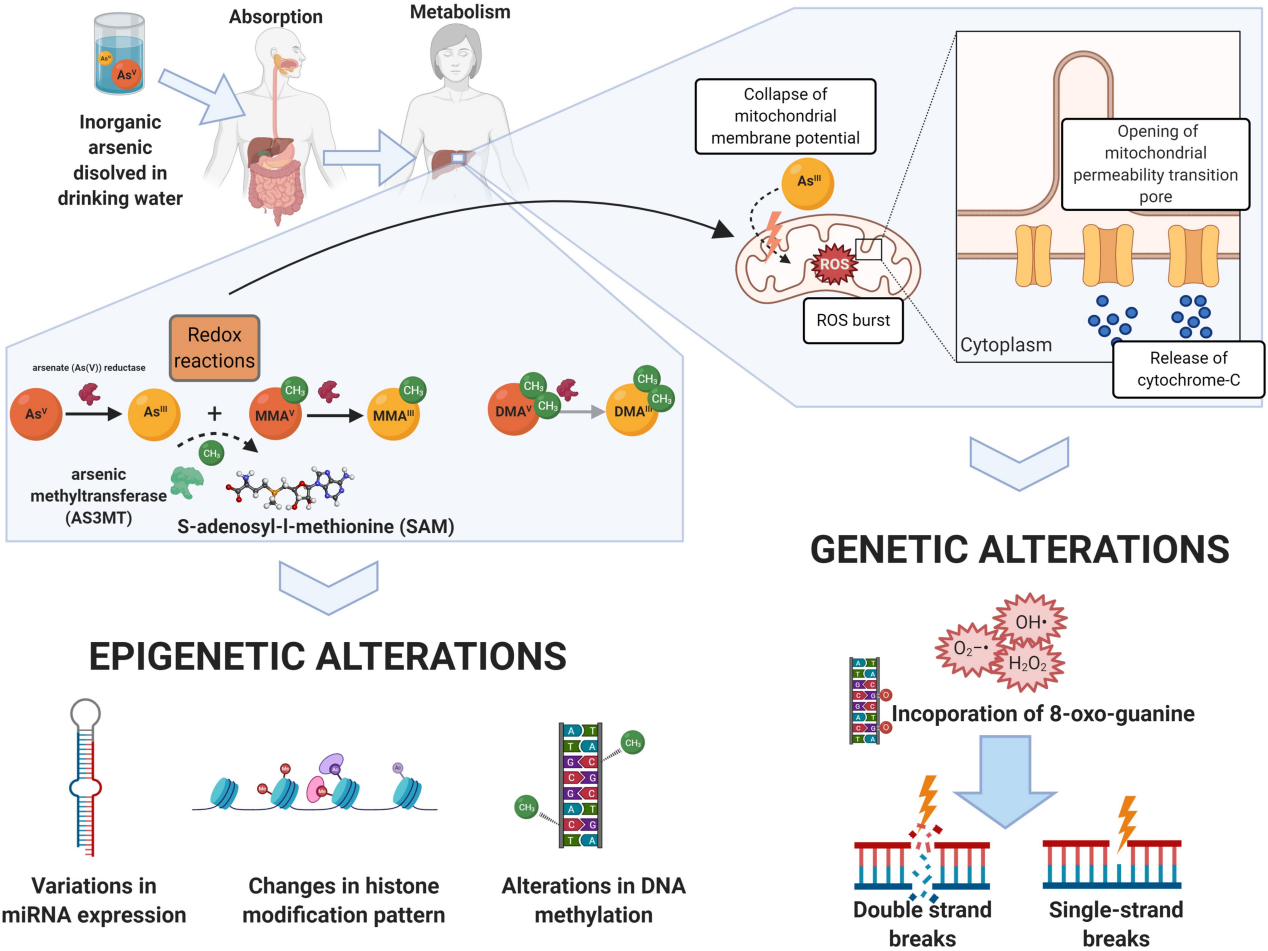
Arsenic biotransformation as mechanisms for molecular damage. Arsenic dissolved in drinking water (mainly as arsenate, in its oxidation state +5, As[V]) is readily absorbed by the gastrointestinal tract. Biotransformation of arsenic occurs in the liver, where arsenite (As[III]) and As[V] goes through a series of redox reactions, resulting in the generation of mono and dimethylated arsenic intermediaries: Monomethylarsonous acid (MMA[III]), monomethylarsonic acid (MMA[V]), dimethylarsinous acid (DMA[III]), and dimethylarsinic acid (DMA[V]). S-adenosylmethionine (SAM) is the methyl donor during arsenic biotransformation to both oxidation state III and oxidation state V arsenic species. The transfer of a methyl group from SAM to trivalent arsenicals is catalyzed by the arsenic6 (+3 oxidation state) methyltransferase (AS3MT), producing methylated and dimethylated arsenicals. Epigenetic alterations, including global and local DNA methylation changes, alteration of miRNA expression, and changes in the histone modification pattern, are associated with this process. On the other hand, the redox reactions involved in arsenic biotransformation lead to ROS-mediated disruption of the mitochondrial electron transport chain, which subsequently results in DNA damage such as double- and single-strand breaks, and specific mutational patterns. Created with BioRender.com.

Arsenic is found in its inorganic forms: arsenite (As[III], oxidation state+3) and arsenate (As[V], oxidation state+5). Arsenate is the main form present in drinking water and is readily absorbed by the gastrointestinal tract (Figure 2). The metabolization occurs in the liver, where As[V] is reduced to As[III], in a reaction catalyzed by the purine nucleoside phosphorylase (PNP) and glutathione-S-transferase omega. Subsequently, additional redox reactions involving As[V] and As[III] occur (Drobna et al., 2009; Minatel et al., 2018).

Sequential addition of methyl groups to the intermediaries generates mono- and dimethylated arsenicals (+3/+5). The arsenic [+3] methyltransferase (AS3MT) catalyzes the transfer of a methyl group from S-adenosylmethionine (SAM) to trivalent arsenicals. This reaction generates monomethylarsonous acid (MMA[III]), monomethylarsonic acid (MMA[V]), dimethylarsinous acid (DMA[III]), and dimethylarsinic acid (DMA[V]; Dopp et al., 2004; Antonelli et al., 2014; see Figure 2). This process facilitates its excretion through urine; however, prolonged exposure (even at low levels) might convert this detoxification process into one of the possible mechanisms inducing genetic and epigenetic alterations linked to arsenic exposure.

Polymorphisms in genes involved in the arsenic biotransformation pathway are considered a major cause of inter-individual variations in the susceptibility to arsenic-related health conditions (reviewed in Paul et al., 2015; Minatel et al., 2018). For example, single nucleotide variants (SNVs) in *AS3MT*, *PNP*, and *GSTO(1/2)* are significantly associated with arsenic-induced dermatological lesions (De Chaudhuri et al., 2008; Paul et al., 2015). It has been proposed that these SNVs could modify physicochemical features of these enzymes particularly in regions close to the arsenic binding site (De Chaudhuri et al., 2008).

## Genetic and Epigenetic Effects Associated with Arsenic Exposure

Genetic and epigenetic mechanisms are involved in arsenic-induced health effects (summarized in Figure 2 and Table 1).

**Table 1:** Genetic and epigenetic molecular changes associated with arsenic exposure.

Molecular effects associated with arsenic exposure	Examples of molecular mechanisms involved		References
	Genetic	Epigenetic	
Generation of reactive oxygen species (ROS)/promotion of oxidative and genotoxic damage	Activation of NADPH oxidase and nitric oxide synthase Disruption of the mitochondrial electron transport chain Chromosome instability (e.g., chromatid breaks, aneuploidy, increased copy number, and presence of micronuclei)	Elevated levels of H3K9me2 within promoter regions of genes involved in the Base excision repair (BER) pathway (MPG, XRCC1, and PARP1)	Barchowsky et al., 1999; Smith et al., 2001; Chakraborty and De, 2009; Cooper et al., 2009; Kitchin and Conolly, 2010; Martinez et al., 2010; Smeester et al., 2011; Roy et al., 2016; Smeester and Fry, 2018; Ding et al., 2021
Interference with DNA repair	Downregulating the expression of genes involved in NER	Hypomethylation of the ERCC2 promoter BRCA1 hypermethylation and decreased BRCA1 and estrogen receptor alpha Promoter hypermethylation/decreased expression of MMR genes (MSH2, MLH1)	Paul et al., 2014; Mauro et al., 2016; Bhattacharjee et al., 2018; Selmin et al., 2019
Activation of proliferative and survival pathways	Arsenic-induced phosphorylation of AKT Increased expression and amplification of KRAS transcript carrying oncogenic mutations Suppression of differentiation through EGFR Mitochondrial-dependent temporal dysregulation of cyclin D1	Increase the expression of miRNA-21 (a well-known cancer-related miRNA) through overexpression of HIF-1α induced by arsenic-mediated activation of the PI3K/AKT pathway	Liu et al., 2012; Cheikhi et al., 2020
Induction of CSC-like properties/EMT	Increased expression of stemness genes Activation of NRF2 and HIF1α Elevated levels of CD34+ cells and expression of RAC1	Promoter hypomethylation of SEPT9 in human colorectal cancer cell lines	Waalkes et al., 2008; Rafiei et al., 2019; Bi et al., 2020, 2021; Chang et al., 2020; Wadgaonkar and Chen, 2021
Alteration of methylation patterns	Inhibition/reduction of the mRNA expression of DNA methyl transferases (DNMT1/2/3A and 3B). Potentially linked to hypomethylation	S-adenosylmethionine (SAM) depletion Global hypomethylation: at LINEs/LTRs retrotransposon and specific gene promoters Telomere lengthening lead by altered subtelomeric methylation pattern/loss of heterochromatinization (H4K20me3 mark)	Reichard and Puga, 2010; Paul et al., 2014; Rafiei et al., 2019; Bhattacharjee et al., 2020; Nohara et al., 2020a
Effects in mitochondria	Stimulates mitochondrial EGFR activation (linked to increased ROS/oxidative damage)	Hypomethylation in D-loop and ND6 gene along with increased expression of ND4, ND6, mtTfam, and higher mtDNA copy number	Sanyal et al., 2018; Cheikhi et al., 2020

Genetic mechanisms associated with arsenic exposure include the promotion of oxidative and genotoxic damage and a decrease in DNA repair (Kitchin and Conolly, 2010; Smeester et al., 2011; Minatel et al., 2018; Smeester and Fry, 2018). At the epigenetic level, arsenic has been shown to induce DNA hypo- and hypermethylation, disrupt the expression of microRNAs (miRNAs) and alter the histone modification pattern (Smeester et al., 2011; Chervona et al., 2012; see Figure 2 or Table 1). Some of these mechanisms have been observed as a response to gestational exposure to arsenic (Pilsner et al., 2012; Nohara et al., 2020b).

### Genetic Effects

Arsenic can act as a co-mutagen with other chemicals and UV light, leading to increased oxidative stress and subsequent DNA damage (Hartwig et al., 1997; Rossman et al., 2004). Additionally, It has been shown that arsenic is associated with a distinctive mutational spectrum in lung squamous cell carcinomas arising in never smokers (Martinez et al., 2013). The toxicity of the trivalent forms of arsenic is higher than the pentavalent species, owing to higher affinity for sulfhydryl groups and cysteine-binding capacity, resulting in loss of protein activity, impaired recruitment, and loss of the protein–protein/protein-DNA interaction capacity (Shen et al., 2013; Moe et al., 2016; Hirano, 2020).

The cycling between As[III] and As[V] generates oxygen-derived radicals, such as superoxide anion (O_2_^•−^), hydroxyl radical (•OH), hydrogen peroxide (H_2_O_2_), singlet oxygen (^1^O_2_), and peroxyl radicals (Halliwell and Whiteman, 2004; see Figure 2). One of the main mechanisms of arsenic-induced ROS generation involves the disruption of the mitochondrial electron transport chain *via* ROS accumulation and/or by inducing permeability transition linked to the high affinity of arsenic for sulfhydryl groups, leading to apoptosis (Pulido and Parrish, 2003; Liu et al., 2005). MMA[III] can also induce the formation of ROS, promoting the formation of DNA adducts, double-strand breaks, and specific mutational patterns (Wang et al., 2001; Kligerman et al., 2010; Naranmandura et al., 2011). DMA[III] seems to be more readily taken up by cells and capable of inducing the formation of micronuclei due to the generation of free radicals (Kenyon and Hughes, 2001; Zamora et al., 2014). The levels of mutagenic oxidative DNA lesions (8-oxo-7,8-dihydro-2'-deoxyguanosine and 8-nitroguanine), DNA strand breaks, and micronucleus frequency in cord blood have been shown to be associated with arsenic exposure *in utero* (Navasumrit et al., 2019).

Arsenic interferes with DNA repair by downregulating the expression of genes involved in the nucleotide excision repair process (Walter et al., 2007; Nollen et al., 2009; Osmond et al., 2010; Minatel et al., 2018). Arsenic also disrupts proliferative and survival pathways (Wang et al., 2014a; Person et al., 2015), such as PI3K/Akt signaling, through arsenic-induced phosphorylation of AKT (Gao et al., 2004; Liu et al., 2012, 2020). Likewise, it has been shown that arsenic can induce cancer stem cell (CSC)-like properties. For example, arsenic promotes a metabolic shift from mitochondrial TCA cycle to glycolysis through activation of NRF2 and HIF1α, a defining characteristic of arsenic-induced CSCs (Bi et al., 2020; Wadgaonkar and Chen, 2021). Arsenic can also preserve a germinative state in cultured human epidermal cells and alters signal transduction related to proliferative potential (Patterson et al., 2005). Fetal arsenic exposure can also increase the effects of skin carcinogens (topical 12-O-tetradecanoyl phorbol-13-acetate, TPA) in mice by elevating the levels of CD34+ cells and expression of RAC1 (involved in self-renewal stimulation; Waalkes et al., 2008). Bronchial epithelial cells exposed to arsenic, acquired CSC-like features such as asymmetric division, self-renewal, and increased expression of stemness genes (Chang et al., 2020; Bi et al., 2021).

Arsenic-mediated malignant transformation has been linked to disruption of the *KRAS* gene. Arsenite-mediated transformation of human prostate epithelia has been linked to increased expression and amplification of *KRAS* transcript carrying oncogenic mutations (G12S and A59T; Merrick et al., 2019). Additionally, long-term arsenite exposure can cause activation of human endogenous retroviruses related to *KRAS* gene fusion and oncogenic amplification, which has been associated with arsenic-mediated epigenetic effects resulting in de-repression of retroviral sequences (Merrick et al., 2020).

### Epigenetic Effects

Prolonged exposure to arsenic can lead to the depletion of SAM. The depletion is likely due to arsenite methylation competing with many cellular processes that require methyl groups provided by SAM, including DNA, RNA, and histone methylation (Simeonova and Luster, 2000; Reichard et al., 2007; Ouyang et al., 2020). Thus, the arsenic-induced depletion of the cellular pool of methyl groups can have an impact on epigenetic reprogramming and thus modify disease susceptibility.

Exposure to arsenic has been linked to both hypo- and hypermethylation affecting gene expression in humans (Bailey and Fry, 2014). Global hypomethylation can be explained by the competition for the pool of SAM-provided methyl groups and by the arsenic-mediated inhibition of DNA methyltransferase (DNMT) enzymes (Reichard and Puga, 2010). In addition to global hypomethylation, arsenic reduces methylation at gene promoters and specific regions of the DNA. Long-term (20days) exposure to low levels (1 and 0.1μM) of inorganic arsenic induces promoter hypomethylation of *SEPT9* in human colorectal cancer cell lines, which is correlated with epithelial-mesenchymal transition (Rafiei et al., 2019). Similarly, arsenic induces hypomethylation of the *ERCC2* promoter both *in vivo* and *in vitro*, leading to *ERCC2* overexpression, inhibition of Cdk-activating kinase complex release, and decrease of p53 phosphorylation (serine 392; Paul et al., 2014). The disruption of ERCC2 and the downstream effects might contribute to increased DNA damage observed among arsenic exposed individuals. On the other hand, arsenic can also decrease methylation levels in specific regions of the DNA harboring non-coding sequences. Long interspersed nuclear element-1 (LINE-1). LINE-1 methylation levels are lower among individuals living in arsenic-endemic areas compared to those in non-endemic areas, particularly among female individuals (Hossain et al., 2017). The mechanisms governing hypomethylation of specific DNA sequences linked to arsenic exposure are not yet fully elucidated. It has been proposed that gestational arsenic exposure can increase hypomethylated cytosines, with accumulation in the promoter regions of the active full-length L1MdA subfamily of LINEs, potentially enhancing retrotransposition and cryptic promoter activity of 5' long-terminal repeats for coding genes and non-coding RNAs (Nohara et al., 2020a). LINE-1 hypomethylation has been associated with cardiovascular disease (CVD; Muka et al., 2016), which is also a hallmark of arsenic-induced chronic conditions. Arsenic also induces global hypomethylation (Alu/LINE-1 methylation status) in children chronically exposed to arsenic (even at low levels), resulting in genotoxic stress (Alegría-Torres et al., 2016; Bandyopadhyay et al., 2016).

The mechanisms underlying arsenic-induced gene-level hypermethylation are still unknown. A positive correlation has been described between the concentration of arsenic in the mother’s urine and global DNA hypomethylation in an infant’s cord blood sample (Pilsner et al., 2012). The effects of prenatal exposure to arsenic on the fetal epigenome have been also observed in a sex-dependent manner. *In utero* exposure to arsenic (measured through maternal urinary arsenic concentration) induced the expression of a completely different set of genes involved in epigenetic pathways in male vs. female fetal placentas (Winterbottom et al., 2019). On the other hand, *in utero* and early life exposure has been associated with increased DNA methylation in the promoter region of extracellular matrix remodeling gene MMP9, accompanied by a reduction of protein levels (Chicoine et al., 2002; Gonzalez-Cortes et al., 2017). Similarly, gestational exposure to arsenic (before the 25th week) has been associated with increased methylation in repetitive sequences, as well as with higher methylation in promoter regions of tumor suppressor genes (*CDKN2A* and *TP53*) in umbilical cord leukocytes (Kile et al., 2012; Cardenas et al., 2015).

Recent studies indicate that exposure to arsenic can affect the methylation status of human mitochondrial DNA (mtDNA). Arsenic is able to induce hypomethylation of the D-loop region (critical for mtDNA replication and transcription) and of the ND6 gene, along with increased expression of ND4, ND6, mtTfam and higher mtDNA copy number in individuals exposed to arsenic in drinking water (Sanyal et al., 2018). Arsenic-induced mitochondrial damage has been also associated with increased ROS/oxidative damage following exposure. For example, exposure to low levels of arsenic stimulates mitochondrial EGFR activation as an upstream mechanism for arsenic dysregulation of reserve cells, increasing mtROS, and proliferative signaling in Murine C2C12 myoblasts (Cheikhi et al., 2020). Other cellular events associated with disruption of the EGFR includes is the suppression of differentiation through EGFR and mitochondrial-dependent temporal dysregulation of cyclin D1 (Cheikhi et al., 2020), which might explain arsenic-mediated impairment of differentiation in different cell types (Macoch et al., 2013). Mechanisms related to low-level exposure differ from those linked to high-level exposure, where mitochondrial ATP generation is inhibited, resulting in cell death (Cheikhi et al., 2020). In children, low to moderate level arsenic exposures (<100ppb) cause strength and motor deficits (Parvez et al., 2011), possibly as the result of targeting of muscle and progenitor cell mitochondria, with disruption of muscle metabolism, maintenance, and regeneration (Ambrosio et al., 2014).

Experimental models have contributed to elucidate the correlation between the epigenetic modifications induced by gestational exposure to arsenic and disease development. The offspring of pregnant mice transplacentally exposed to arsenic in drinking water showed a sex-dependent increase in tumor incidence after 74 (males) and 90weeks (females) compared with non-exposed mice (Waalkes et al., 2003). Promoter hypomethylation of genes involved in estrogen signaling with the concurrent increase in mRNA expression has been observed in hepatic tumors in adult mice exposed to arsenic *in utero* (Waalkes et al., 2004). Changes in the histone modification pattern, including an increase in H3K4 trimethylation in the promoter region of *Fatty acid binding protein 4* (*Fabp4*) and genome-wide hypo-acetylation at H3K9, have been observed in brain samples of the offsprings of mice prenatally exposed to arsenic, compared to non-exposed mice (Nohara et al., 2012; Cronican et al., 2013). Exposure to arsenic can increase the expression of miRNA-21 (a well-known cancer-related miRNA) through overexpression of HIF-1α induced by arsenic-mediated activation of the PI3K/AKT pathway (Liu et al., 2020). The expression of other miRNAs involved in apoptosis (miR-143), cell proliferation/migration (miR-200b and miR-222), and apoptosis (miR-27a), is also disrupted by exposure to inorganic arsenic (Wang et al., 2014b, 2016; Ngalame et al., 2016; Zhang et al., 2016). Moreover, *in utero* arsenic exposure (measured in cord blood) strengthens the negative association between miR-1290 and birthweight in a cohort of mother-infant pairs enrolled in a prospective birth cohort in Bangladesh (Rahman et al., 2018).

## Gestational Exposure to Arsenic and Increased Risk of Disease

Fetal and early postnatal stages of development are particularly sensitive to the impact of environmental exposure to arsenic. Such exposures may compromise early developmental processes and predispose the fetus to adverse health risks later in life (Barker, 2007; Grandjean et al., 2015). Furthermore, changes to the phenotype in the following generations can be observed even in the absence of direct environmental exposures (Skinner, 2011; Nilsson et al., 2018).

The intrauterine environment to which the fetus is exposed is regulated by the placenta, a fetomaternal organ that regulates critical aspects of embryonic development and pregnancy, including immune responses, gas and nutrient transfer, endocrine function, and protection from environmental exposures (Carter, 2015; Wilson and Robinson, 2018). Arsenic compounds can cross through the placental barrier, so exposure in pregnant women is significant for fetal development (Ramsey et al., 2013). The concentration of arsenic found in the human placenta correlates with both maternal and infant levels, as well as with the levels in the household drinking water (Punshon et al., 2015).

Increasing evidence suggests that gestational exposure to arsenic affects fetal development and induces disease phenotypes that develop only later in life (Nohara et al., 2020b). In Northern Chile, the levels of arsenic in drinking water increased from about 90 to 870ppb in 1958 (Romero et al., 2003; Thomas, 2013). In the 1970s, remediation measures reduced levels to approximately 110ppb, generating two cohorts of individuals exposed to significantly different levels of arsenic (Smith et al., 2012). The adult mortality for individual types of cancer (urinary bladder, respiratory tract, kidney, and liver), and all cancer types combined was significantly higher in the cohort exposed to the highest levels of arsenic in drinking water (born between 1958 and 1970; Smith et al., 2012). A study analyzing childhood cancer mortality for the same region found an increased risk in childhood (ages 10–19) of liver cancer mortality (Liaw et al., 2008).

Arsenic-related health effects resulting from gestational or early life exposure to arsenic in drinking water have been also observed in CVDs and other conditions. For example, early-life (3–8years old) exposure to inorganic arsenic was significantly associated with higher values of blood pressure and left ventricular mass (a predictor of adverse cardiovascular events and premature death) and with lower ejection fraction (indicating impaired contraction) in Mexican children exposed to arsenic concentrations in drinking water between 3 and 398ppb (Osorio-Yáñez et al., 2015). Additionally, early life exposure appears to have gender-specific effects among the pediatric population. In a rural population in Bangladesh, young girls (12–18years old) were at increased risk of mortality from all causes (Rahman et al., 2013). Furthermore, *in utero* exposure has been also associated with increased susceptibility to impaired thymic and lung function (Dauphiné et al., 2011; Ahmed et al., 2012), body size (Saha et al., 2012), and motor function (Parvez et al., 2011). A similar situation was observed for bronchiectasis and other chronic obstructive pulmonary disease, acute myocardial infarction, chronic renal disease, and all non-cancer causes of death (Thomas, 2013; see Figure 1).

The role of arsenic in the development of other significantly associated conditions, such as CVD, are also closely related to its biotransformation. Increase in ROS generation can result in disturbance of endothelial function (inhibition of endothelial nitric oxide synthase) leading to lipid peroxidation, which is one of the most commonly proposed mechanisms in arsenic-induced CVD (Barchowsky et al., 1999; Liao et al., 2006; Sidhu et al., 2015). Moreover, exposure to inorganic arsenic through drinking water induces pathological hypertrophy of the heart in male rats by activating the calcineurin-nuclear factor of activated T cells (NFAT) signaling pathway (Kabir et al., 2021).

## Discussion

Several studies show that arsenic exposure at different stages of life has detrimental effects on human health, both in the short term (prenatal, perinatal, and early childhood), as well as later in life (increased risk of developing diseases). Effects during fetal development and early life stages are defined by the dynamics of the transit of arsenic across the placental barrier. Factors such as concentration, length of exposure (mother), and effects of the genotype might influence significantly the health effects associated with gestational exposure. Additionally, the effects during fetal development should consider that the organs responsible for arsenic metabolism are not fully developed yet (full maturity of the liver takes up to 2years after birth; Beath, 2003). Therefore, the metabolism of arsenic during the gestational/postnatal period could present some differences compared to the established model in a fully-functional liver.

The precise mechanisms underlying the long-term arsenic-related effects are yet to be elucidated. Arsenic-related epigenetic alterations seem to play a significant role in these effects, suggesting that imprinting could be involved (Smeester et al., 2011; Bailey et al., 2013; Sanders et al., 2014). Moreover, arsenic-induced epigenetic alterations of imprinted genes (such as Igf2 and H19) generated during prenatal exposure can have deleterious effects (spermatotoxicity) in male offsprings of subsequent generations (Yin et al., 2021). However, how these alterations could impact fetal development and drive long-term tumorigenesis is still unknown.

One possible explanation for the arsenic-induced long-term effects is the cumulative molecular damage, as a result of chronic exposure. While some of the early molecular alterations induced by arsenic could be repaired, others (particularly epigenetic) could persist and progressively generate a favorable scenario for disease-prone alterations. The alterations identified in the adult population might already reflect some degree of molecular damage accumulation. In contrast, alterations identified during early stages of development could represent the first indications of arsenic-induced molecular damage. Thus, the identification of specific genetic/epigenetic alterations linked to gestational arsenic exposure would contribute to monitoring those individuals at higher risk of developing health effects during childhood, as well as later in life.

Cohorts of exposed human populations have been pivotal in identifying some of the health outcomes linked to early arsenic exposure; however, it is difficult to use these cohorts to identify the series of events occurring over time, particularly at a molecular level. Identifying the sequence of molecular events involved in arsenic-induced molecular damage remains as one of the critical gaps in our understanding of the modes of action of this environmental compound. Clues to decipher early events in arsenic-induced disease could be obtained from longitudinal *in vitro* models, where exposure at physiologically-relevant doses could be mimicked. Such models could potentially generate the preliminary information that could be validated in *in vivo* models.

Importantly, the combined use of new technologies will help to elucidate an extended network of biological effects associated with arsenic exposure. So far, molecular events associated with arsenic exposure have been analyzed individually. The incorporation of different omics technologies in the analysis of these longitudinal *in vitro* cohorts will help to elucidate how changes at DNA and RNA level are acting concertedly. For example, the use of next generation sequencing technologies will help to interpret methylation and other modification in DNA and how these changes can influence changes in the levels of messenger RNAs, as well as of long and short non-coding RNAs. Ultimately, integrating these molecular events will contribute to the development of integrated genetic and epigenetic-based signatures as biomarkers of the cumulative effects of arsenic exposure that could lead to early detection of arsenic-related diseases.

## Author Contributions

VM is the principal and corresponding author, and compiled and analyzed the literature on arsenic-related health effects. VM and WL designed the mini-review article. All authors contributed to the article and approved the submitted version.

## Funding

This work was supported by grants from the Canadian Institutes of Health Research (FDN-143345) and National Institutes of Health (R01HD089713), and funds from the BC Cancer Foundation.

## Conflict of Interest

The authors declare that the research was conducted in the absence of any commercial or financial relationships that could be construed as a potential conflict of interest.

## Publisher’s Note

All claims expressed in this article are solely those of the authors and do not necessarily represent those of their affiliated organizations, or those of the publisher, the editors and the reviewers. Any product that may be evaluated in this article, or claim that may be made by its manufacturer, is not guaranteed or endorsed by the publisher.
