# Supplementary figures and images for: Health Effects Associated With Pre- and Perinatal Exposure to Arsenic
Source: Front Genet. 2021 Sep 29;12:664717. doi: 10.3389/fgene.2021.664717 (PMC8511415; doi:10.3389/fgene.2021.664717)

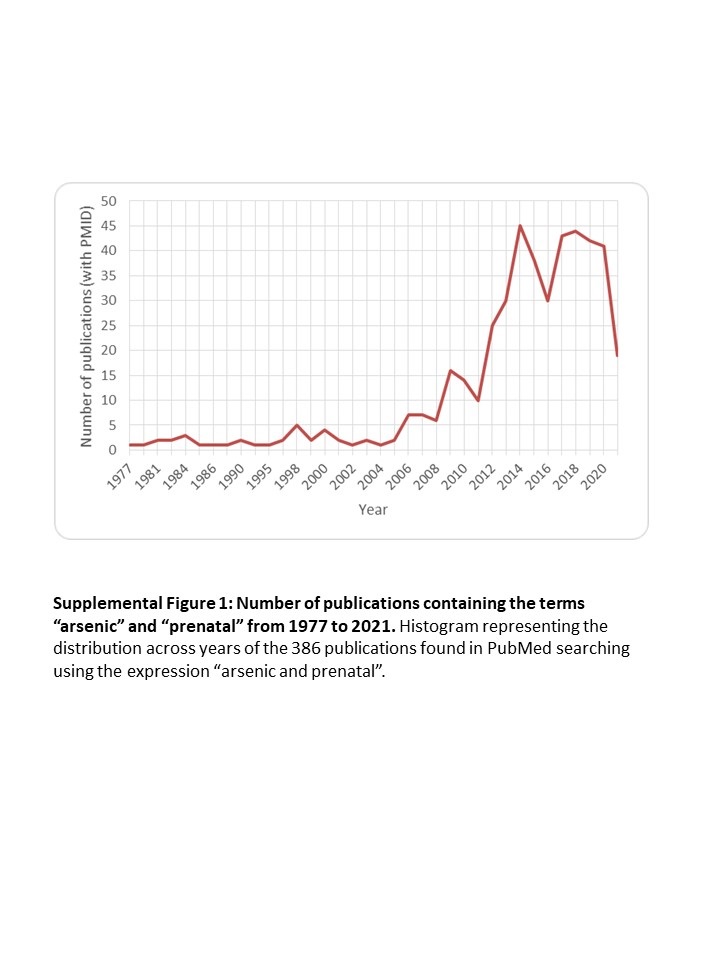

Supplement: Supplementary file 1 [file Image_1.jpeg]
